# Radiomics in radiation oncology—basics, methods, and limitations

**DOI:** 10.1007/s00066-020-01663-3

**Published:** 2020-07-09

**Authors:** Philipp Lohmann, Khaled Bousabarah, Mauritius Hoevels, Harald Treuer

**Affiliations:** 1 Institute of Neuroscience and Medicine (INM-4), Research Center Juelich, Wilhelm-Johnen-Str., 52428 Juelich, Germany; 2 Department of Stereotaxy and Functional Neurosurgery, Center for Neurosurgery, Faculty of Medicine, University Hospital Cologne, Kerpener Str. 62, 50937 Cologne, Germany

**Keywords:** Artificial intelligence, Machine learning, Deep learning, Textural features, Radiotherapy, Multiparametric positron emission tomography/magnetic resonance imaging (PET/MRI)

## Abstract

Over the past years, the quantity and complexity of imaging data available for the clinical management of patients with solid tumors has increased substantially. Without the support of methods from the field of artificial intelligence (AI) and machine learning, a complete evaluation of the available image information is hardly feasible in clinical routine. Especially in radiotherapy planning, manual detection and segmentation of lesions is laborious, time consuming, and shows significant variability among observers. Here, AI already offers techniques to support radiation oncologists, whereby ultimately, the productivity and the quality are increased, potentially leading to an improved patient outcome. Besides detection and segmentation of lesions, AI allows the extraction of a vast number of quantitative imaging features from structural or functional imaging data that are typically not accessible by means of human perception. These features can be used alone or in combination with other clinical parameters to generate mathematical models that allow, for example, prediction of the response to radiotherapy. Within the large field of AI, radiomics is the subdiscipline that deals with the extraction of quantitative image features as well as the generation of predictive or prognostic mathematical models. This review gives an overview of the basics, methods, and limitations of radiomics, with a focus on patients with brain tumors treated by radiation therapy.

## Introduction

The diagnosis of brain tumors and the assessment of response to radiotherapy [1–4] are mainly based on the results of modern neuroimaging techniques and, essentially, the histomolecular examination of tissue samples collected during tumor resection or biopsy. For decades, brain tumor patients have been diagnosed by means of structural neuroimaging techniques such as contrast-enhanced computed tomography (CT) or magnetic resonance imaging (MRI). In recent years, advanced imaging methods have entered clinical routine. These include, in particular, perfusion (PWI)- and diffusion-weighted (DWI) MRI, as well as amino acid positron emission tomography (PET) [5, 6]. In combination with the anatomic information, these methods provide functional and metabolic parameters that are of great benefit in the assessment of, e.g., treatment response or estimation of prognosis. The increasing availability of hybrid PET/CT and PET/MRI scanners also simplifies the use of these advanced techniques in a clinical setting. However, with the increasing amount of data available for diagnosis, a complete, accurate, and timely evaluation of the data in clinical routine is almost impossible without considerable computer support.

Since methods from the fields of artificial intelligence (AI) and machine learning allow for a partial or full automation of various steps within the diagnostic routine, it is not surprising that these methods are investigated extensively and have already been applied in clinical routine in some cases. Especially in the field of radiotherapy, the automated detection of lesions such as brain metastases and the subsequent segmentation is of importance. These methods not only support the radiation oncologist, resulting in increased productivity, but can, in addition, help to detect small lesions which are frequently overlooked otherwise. Furthermore, computer-aided analysis of the large amount of information obtained from structural and functional neuroimaging may also help to increase the comparability of results as it does not depend on the experience level of the evaluating clinician.

Besides automation of laborious clinical procedures such as the manual detection and segmentation of lesions for radiotherapy planning, AI also offers the potential to extract otherwise undiscovered features from the imaging data for diagnostic use. These quantitative imaging features further characterize the underlying tumor biology and are usually beyond human perception. These features can be combined with conventional imaging parameters from structural and molecular neuroimaging as well as with clinical data such as the patient’s age or molecular markers to develop predictive or prognostic mathematical models that are subsequently used to answer clinical questions, such as the assessment of treatment response to radiation therapy or the non-invasive diagnosis of molecular parameters [7]. The extraction of quantitative imaging features as well as the generation and evaluation of mathematical models for diagnosis is termed *radiomics* and can be regarded as a special application of AI [8–12].

However, the use of these computer-based methods must be carefully and critically evaluated. The applied neuroimaging protocols and AI-based methods are poorly standardized and vary substantially. This review article gives an overview of the basics, methods, and limitations of radiomics, with a special focus on feature-based radiomics in brain tumors treated by radiation therapy.

## Radiomics

Radiomics aims at the extraction of quantitative parameters from routinely acquired medical imaging data, thereby allowing additional data analysis at low cost. Most of the features characterize the underlying image (tumor) heterogeneity. In classic radiomics approaches, sometimes also called *feature-based radiomics*, the radiomics features to be extracted are predefined and calculated from a manually or semi-automatically segmented image. In contrast, *deep learning-based radiomics* follows a different approach for the extraction of quantitative parameters. Here, the radiomics features are not predefined, but identified and generated from the underlying data by computational models. Furthermore, image segmentation is not necessarily required for deep learning-based radiomics, despite providing image segmentations usually improves model performance. Although most of the radiomics models have to prove their value in the clinical setting, the process of feature extraction applies semi- or fully automated methods from advanced statistics and machine learning and may as such lead to more robust, reproducible, and reliable results compared to the reader-dependent clinical interpretation of imaging data.

### Feature-based radiomics

To calculate radiomics features, manual or semi-automatic segmentations of the region of interest (ROI) or volume of interest (VOI) are mandatory. Typically, the contrast-enhancing portion of the tumor in MRI is used for radiomics analysis. However, including information contained in the infiltration zone of the lesion by also considering signal abnormalities on T2-weighted or fluid-attenuated inversion recovery (FLAIR) MRI provides a more realistic representation of the whole tumor and allows the radiomics analysis to be performed on a larger segment, potentially encoding more information and resulting in a better diagnostic performance. Although the number of studies using amino acid PET images for radiomics analysis is still low, especially the combined analysis of amino acid PET and MRI radiomics encodes more diagnostic information than either modality alone [13, 14] and might gain clinical relevance. In patients with brain tumors, image segmentation in clinical routine is usually performed manually on CT or MRI for the purpose of radiotherapy planning or volumetric assessment of therapy response. However, a manual, three-dimensional differential segmentation of tumor regions with contrast enhancement, necrosis, and perifocal edema is laborious, time consuming, and strongly dependent on the performing physician. Methods from the field of AI including textural feature analysis and deep learning-based methods are already available and currently under investigation for routine clinical application [15–21].

As mentioned above, radiomics aims at the extraction of quantitative imaging features from routinely acquired imaging data. Consequently, in order to enable a high reproducibility and generalizability of the results, the image data have to undergo several *preprocessing* steps before feature extraction. One of the first preprocessing steps is *interpolation* of the imaging data to isotropic voxel spacing, which allows for a better comparison of heterogenous, multi-institutional imaging data. Furthermore, the calculation of radiomics features, especially textural features, requires rotationally invariant voxels, achieved by interpolation. Images can either be upsampled, e.g., the original image with a voxel spacing of 1.0 × 1.0 × 3.0 mm^3^ is upsampled to 1.0 × 1.0 × 1.0 mm^3^, or downsampled, e.g., the original image with a voxel spacing of 1.0 × 1.0 × 3.0 mm^3^ is downsampled to 3.0 × 3.0 × 3.0 mm^3^. While upsampling introduces artificial information and might increase image noise, downsampling conversely incurs information loss. Consequently, there is currently no clear recommendation for either of the two procedures [22].

*Discretization* or quantization of image intensities is of particular importance to allow for comprehensible feature extraction [22]. Two methods for image discretization are commonly used. The first method performs a discretization of the image intensities to a *fixed number of bins*, which allows for a direct comparison of feature values across different patients and partly performs an image normalization, which is of importance for imaging procedures such as structural MRI that are usually acquired in arbitrary units. However, no correlation to the original image intensities can be established. The second method uses a *fixed bin size*, whereby a new bin is assigned for every intensity interval with a fixed bin with. Importantly, for structural MRI data with arbitrary intensity units, the fixed bin size discretization is not recommended. However, as the relationship to the original intensity scale is maintained, the fixed bin size discretization could be especially useful for quantitative imaging modalities such as PET. Of note, the image discretization has a substantial impact on the extracted radiomics features and, hence, on the reproducibility of the results [22].

Normalization of the image intensities ensures a better comparability of the results between different scanners, protocols, and patients. Commonly used procedures for image intensity normalization are white-stripe [23] or z‑score normalization [24]. Other typical preprocessing steps include, but are not limited to, spatial smoothing, noise reduction, spatial resampling, brain extraction, and corrections of MRI field inhomogeneities.

Following image preprocessing and segmentation, radiomics features can be calculated, most of which reflect tumor heterogeneity. Since the radiomics features are mathematically predefined and based on a huge number of slightly different mathematical definitions, a large number of radiomics features (usually more than 1,000) can be extracted from medical images. Typically, radiomics features are divided into the following subgroups:

i. *Shape features:* quantify the geometric relations and properties of the segmented lesions such as volume, maximum surface area, maximum diameter, compactness, or sphericity [25].
ii. *Histogram-based features *or *first-order statistics: *the distribution of image intensity values within the segmented lesions is typically represented by histograms. From the histograms, quantitative features can be calculated that do not consider any spatial orientation or relationship of the voxels such as the mean, maximum, minimum, median, skewness, or kurtosis [9].
iii. *Textural features *or *second-order statistics: *textural features represent the statistical relationship between the intensity levels of neighboring pixels or voxels or groups of pixels or voxels within the segmented lesion and, thereby, quantify image heterogeneity. Textural features are not extracted directly from the images but from several descriptive matrices that already encode specific spatial relations between pixels or voxels in the segmented lesion. The most commonly used matrices for calculation of textural features are the gray-level run-length matrix (GLRLM), which encodes the size of homogenous runs for each image intensity [26], the neighborhood gray-level different matrix (NGLDM), which corresponds to the difference of intensity levels between one voxel and all of its neighbors in three dimensions, and the gray level co-occurrence matrix (GLCM) [27], which represents the frequency of occurrence of two intensity levels in neighboring pixels or voxels within a specific distance along a fixed direction. Several other matrices exist that encode certain aspects of spatial relations between image intensities in the segmented lesion and, thus, allow the computation of a large number of textural features [27].
iv. *Higher-order statistics features*: the three previous subgroups of features are all usually calculated on the preprocessed original image without any additional image filters. Higher-order statistics features are computed after the application of specific mathematical transformations or filters that aim at highlighting certain aspects of the segmented lesion such as repeating patterns, edges, histogram-oriented gradients, or local binary patterns. Typical mathematical transformations used for the extraction of higher-order statistics features are wavelet or Fourier transforms, fractal analysis, Minkowski functionals, or the Laplacian transform of Gaussian-filtered images (Laplacian of Gaussian) [28].

In summary, a large number of features can be calculated from a single segmented lesion, leading to the problem of distinguishing the parameters relevant to the clinical problem under investigation from the irrelevant and redundant ones. This so-called *feature selection* is of high importance for generating a meaningful predictive or prognostic model from the computed features, especially if the number of available datasets is limited. Feature selection uses advanced statistical methods to identify a subset of features that are neither redundant, constant, duplicated, irrelevant, nor highly correlated [12].

It should be noted that improper feature selection can also lead to *overfitting*, i.e., if a very homogenous dataset from the same scanner acquired with the same protocol is used for feature selection, the features identified as relevant in this particular setting may not be relevant in other settings. Here, overfitting denotes the generation of a model that corresponds too closely or exactly to a particular set of data, whereby it fails to reliably predict outcomes from so far unseen observations. One way to overcome this limitation is to perform feature selection on multicenter datasets ideally representing a large variety of scanners and acquisition protocols, whereby the probability of selecting only locally relevant features, hence, the risk of overfitting is reduced. Unfortunately, in most studies, large multicenter datasets are not available.

Generally, two types of feature selection techniques are used in radiomics studies, *unsupervised* and *supervised* feature selection [29]. Unsupervised feature selection methods such as principal component analysis (PCA) or cluster analysis aim at the identification and removal of redundant features from the feature space, whereby class labels are not considered [12]. Supervised feature selection techniques also take into consideration the relation of the features to the class labels, i.e., features that contribute most to the diagnostic problem are preferred. Consequently, supervised feature selection techniques usually result in better feature subsets compared to the unsupervised methods. Different unsupervised feature selection methods exist, of which *filter methods, wrapper methods*, and *embedded methods* are those most prominent in radiomics.

*Filter methods* are also called univariate methods and statistically evaluate the relation between the features without considering their correlations and interactions. Univariate methods are, for example, the chi-squared score, the Student’s t‑test, the Wilcoxon rank sum test, or the Fisher score [30, 31].

*Wrapper methods*, also called multivariate methods, partly overcome the limitations of univariate methods by taking into consideration correlations and interactions among the radiomics features. While univariate methods only investigate the statistical relationship between the radiomics features, wrapper methods create a subset of features, apply this subset to a predictive model, and evaluate the quality of its performance. Thereafter, a new subset of features is tested and, finally, the best performing subset of features represents the final set of selected features. Due to the iterative nature of wrapper methods, these methods are computationally intensive. These methods include bidirectional search, exhaustive feature selection, forward feature selection, or backward feature elimination [30, 31].

*Embedded methods* combine the advantages of filter and wrapper methods. The feature selection process is performed during the generation of the machine learning model, i.e., during the training phase of the model. Here, interactions and correlations of the radiomics features are considered, leading to more accurate feature selection results compared to filter methods. Additionally, since feature selection is performed during the training phase of the machine learning models and does not require an additional predictive model solely for performance evaluation of the different subsets of features, embedded methods are computationally faster than wrapper methods and less prone to overfitting. Examples of embedded feature selection methods are tree-based algorithms such as the random forest classifier, the least absolute shrinkage and selection operator (LASSO), or ridge regression [30, 31].

Now that a subset of relevant features with low redundancy has been identified by feature selection, a mathematical model for the prediction of a known, underlying ground truth can be generated. This step within the radiomics workflow is called *model generation*. Usually, several different machine learning algorithms and classifiers can be used to generate predictive or prognostic models according to the goal of the study. Among the machine learning algorithms and classifiers most commonly used for radiomics analysis are linear and logistic regression, decision trees, e.g., random forests, neural networks, support vector machines, or the Cox proportional hazards model in case of censored survival data. Model generation includes the iterative search for a set of optimal parameters that define the general structure of the model, a process called *hyperparameter tuning*. In order to identify the best possible machine learning algorithm for the diagnostic problem, the selected model has to be evaluated on a subset of data. To avoid the risk of overfitting by generating and testing the mathematical model on the same subset of data, the available dataset is ideally subdivided into a training and a validation dataset. If the model was trained and evaluated on the same subset of data, a perfect classification of results could be easily achieved by an algorithm that simply repeats the labels of the training data. Obviously, such a highly overfitted model would not provide any useful prediction on new data that was not part of the training dataset. It should be noted, that the distribution of samples of each class remains approximately the same after data splitting, i.e., if 40% of samples were diagnosed with a recurrent tumor, and 60% were diagnosed with treatment-related tissue changes in the original dataset, this proportion of diagnoses should also remain approximately the same in the training and the validation dataset *(stratified split of data)*, which is particularly important for small datasets. Ideally, the model that showed the best diagnostic performance in the validation dataset is finally applied to a test dataset. The test dataset represents the data the model would face when applied in clinical routine. Consequently, every radiomics model should prove its performance, robustness, and reliability on the test dataset, but the test dataset should never be used for tuning of  model hyperparameters.

The described workflow for model generation and evaluation including splitting of the data into three subgroups obviously requires large datasets. Unfortunately, oncological studies including studies in the field of radiation oncology usually contain a maximum of several hundred datasets. However, machine learning also offers methods such as bootstrapping or cross-validation to assess model performance even without the availability of a test dataset. In cross-validation, e.g., 10-fold cross-validation, the dataset is partitioned into ten subsets of equal size. The generated model is evaluated on nine datasets as training data while one subset of data is retained for model validation. This process is repeated ten times, with each subset used once as validation data. Finally, to assess the overall model performance, the classification accuracy from each iteration is averaged.

### Deep learning-based radiomics

Deep learning generally describes the process of applying deep neural network architectures for problem solving, which are particular types of machine learning algorithms. Originally, artificial neural networks that provide the basis for more advanced architectures were inspired by the working principle of the human visual system. By adding layers of hidden neurons beyond the simple input and output layers from artificial neural networks, a new level of complexity was added that enabled deep learning. Deep learning-based radiomics automatically identifies and extracts high-dimensional features from the input images at different levels of scaling and abstraction, resulting in models especially useful for pattern recognition or classification of high-dimensional non-linear data [32]. The usefulness of deep learning-based methods for the automated identification and segmentation of brain metastases and gliomas for radiation therapy planning has been demonstrated in several studies [17–21].

Deep learning-based radiomics uses a workflow that is very different from the feature-based radiomics approach described above. Instead of using mathematically predefined features, different architectures of neural networks such as convolutional neural networks (CNNs) or auto-encoders are used to generate and identify the most important features from the input data. In particular, autoencoder networks, which are a special, unsupervised variant of CNNs, aim at compression of the image content and mapping onto a relatively short but representative feature vector [33]. In general, deep learning-based radiomics uses a cascaded system of single-layer neural networks which are trained to learn and identify structures of relevance for the classification problem in the imaging data [34]. Here, previous mathematical definitions of features and feature selection become unnecessary. Further combinations of the extracted feature vectors are then combined to generate features with an even higher level of abstraction. In a final step, the identified features can be used for classification by the neural network itself, or leave the network and undergo the process of model generation similar to the feature-based radiomics approaches described above using different classifiers from conventional machine learning such as decision trees, regression models, or support vector machines. Of note, while feature-based radiomics always requires segmented images for feature extraction, deep learning-based radiomics, especially CNNs, also function on unsegmented images.

Since the features are generated and extracted directly from the underlying data and a subset of best performing features is automatically extracted, feature selection is rarely performed. However, in order to reduce the risk of overfitting, regularization techniques and dropout of learned connection weights are used. One limitation of deep learning-based radiomics is the high correlation between the features and the input data, as the features are generated from that very data. Therefore, in contrast to feature-based radiomics, large datasets are necessary to identify a relevant and robust feature subset. However, using a machine learning technique called *transfer learning*, this limitation can be partly overcome. In transfer learning, a neural network is used that has already been pre-trained on a different, but closely related task, e.g., a neural network for brain tumor segmentation that was originally trained on imaging data from patients with brain metastases might also provide useful results for the segmentation of glioma patients [35]. Hereby, both the amount of data necessary to identify a relevant feature subset and the computational demand are reduced.

## Limitations

Despite the promising results and the potential of radiomics, the repeatability, reproducibility, and transferability of radiomics features is still an issue and often depends on the used imaging modality, sequence, spatial resolution, size of the image, image quality, reconstruction and correction parameters, as well as motion artefacts and other factors. *Repeatability* is commonly assessed by the extraction of radiomics features from repeated acquisitions of images under identical or near-identical acquisition and processing parameters. In contrast, *reproducibility* of radiomics features, also called robustness, is measured if the acquisition parameters and applied measuring systems differ [36]. A recent review performed an extensive literature search and identified radiomics features that were shown to be repeatable and reproducible among the investigated studies [37]. The authors describe that first-order features, i.e., histogram-based features, were more reproducible than shape metrics and textural features. The most stable feature according to this review was the first-order feature *entropy* [37]. Such systematic review articles focusing on the repeatability and reproducibility of radiomic features are still scarce but of major importance for advancing toward a better standardization of the results from radiomics studies. The Image Biomarker Standardization Initiative (IBSI) provides image biomarker nomenclature and definitions, reporting guidelines as well as benchmark datasets and benchmark values, to enable study groups working in the field of radiomics to verify their image processing and feature extraction [22].

In terms of repeatability and reproducibility, deep learning-based radiomics may be advantageous, as the self-learning neural networks show a better capability for generalization and transfer than feature-based approaches. However, also the models developed from deep learning-based radiomics ultimately have to prove their reliability in clinical routine. Most importantly, data acquisition and analysis as well as model generation need further standardization in order to allow for a better understanding and reproducibility of published results. Although different open-source software packages for feature-based radiomics, (PyRadiomics [38], MaZda [39], and LifeX [40]), as well as open-source frameworks for deep learning-based radiomics (Keras [41], TensorFlow [42], PyTorch [43]) are available, the workflow used in most studies is still complex and often not reported in sufficient detail, so that it is almost impossible for other research groups to fully comprehend the presented results, not to mention reproducing them.

Another limitation to the application of radiomics models in clinical routine is the problem of *interpretability* of the extracted features and the generated models. Mostly, radiomics analysis are perceived as a “black box”, i.e., it is very difficult to (clinically) interpret the generated predictions [36]. However, some methods to improve the interpretability of radiomics analyses have been developed, such as graph-based approaches for feature-based radiomics [44] or visualization tools for deep learning-based radiomics that highlight regions of the segmented tumor according to their importance for the prediction of the generated classifier [45].

Efforts to overcome the mentioned shortcomings are ongoing [22, 46, 47].

## Conclusion

The number of studies evaluating the potential of feature-based as well as deep learning-based radiomics for application in radiation oncology is increasing. Especially in combination with established sources of diagnostic information such as clinical, histomolecular, or conventional imaging parameters, radiomics may contribute significantly towards an improved diagnosis and treatment management in patients with brain tumors and other solid tumors. In radiation oncology in particular, radiomics has great potential for the automated detection and segmentation of target volumes, the differentiation of radiation-induced tissue changes from actual tumor recurrences, and the prediction of the location and timing of local recurrences.
